# Supplementary material for: Monitoring RNA restructuring in a human cell-free extract reveals eIF4A-dependent and eIF4A-independent unwinding activity
Source: J Biol Chem. 2023 Jun 17;299(7):104936. doi: 10.1016/j.jbc.2023.104936 (PMC10362145; doi:10.1016/j.jbc.2023.104936)
Supplement: Supporting Figures S1–S4 [file mmc1.docx]

## Mattie H. O’Sullivan and Christopher S. Fraser

## Supplementary Figure 1


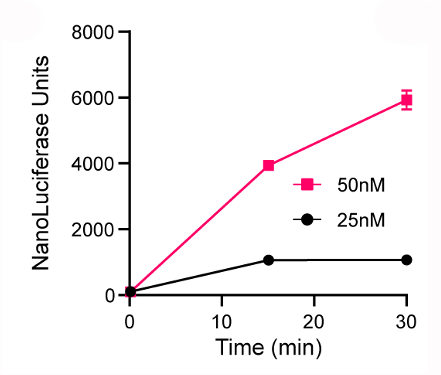


### Figure S1. Optimization of translation conditions in nuclease treated cell-free extract. Line graph depicting luciferase translation of mRNA reporter measured over 30 min at an incubation temperature at 30 °C. *In vitro* translation reactions in lysate programmed with 25 nM or 50 nM mRNA reporter. The lysate was pre-incubate for 10‑minutes prior to initiating protein synthesis by the addition of mRNA, as described in *Experimental Procedures*. Data are presented as means of three independent experiments ± SEM.

##
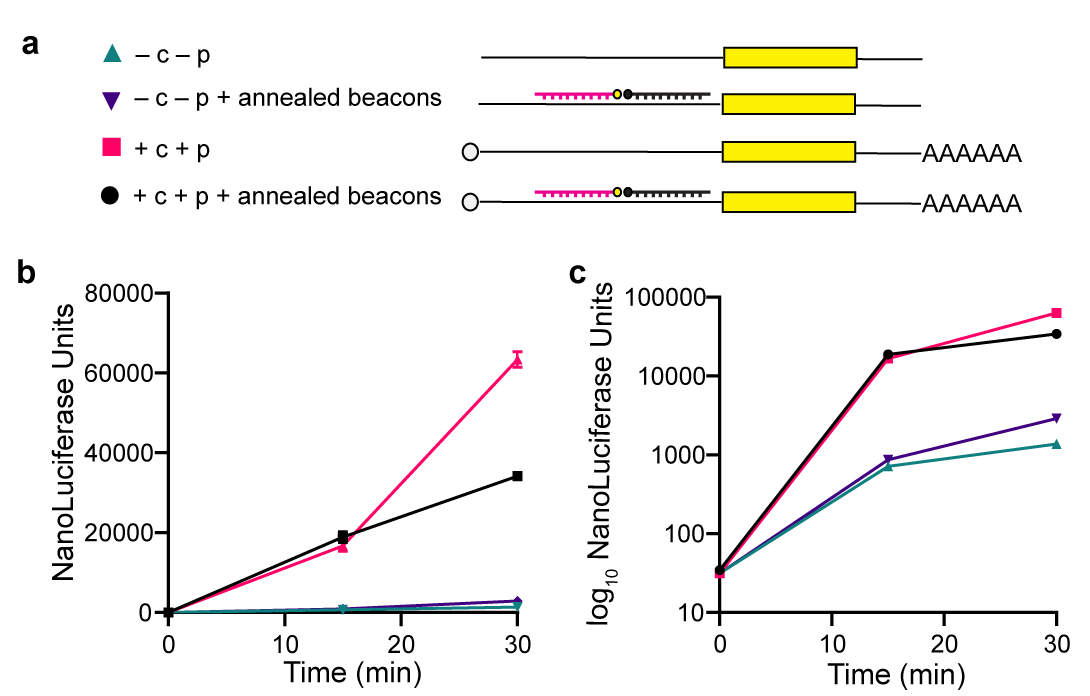
Supplementary Figure 2

## Figure S2. Effect of annealed beacons on protein synthesis. (a) Schematic representation of the reporter constructs used in this set of experiments indicating the combination of m^7^G cap and poly(A) tail and fluorescent beacons annealed to the mRNA 5′ UTR. (b) Line graph depicting luciferase translation of mRNA reporter measured over 30 min at an incubation temperature at 30 °C. *In vitro* translation reactions in lysate programmed with 50 nM mRNA reporter in the absence or presence of m^7^G cap, poly(A) tail, and annealed fluorescent beacons. (c) Line graph using data from (b) but with the Y-axis transformed to a log_10_ scale. All data are presented as means of three independent experiments ± SEM.

**Supplementary Figure 3**

Dual-assay mRNA Reporter Sequence

Parental Plasmid (pUC57) that includes the Globin 5'UTR; 24nt-CY3; 19nt-BHQ, NLuc ORF.

GGTACC: KpnI restriction site.

T7 promoter between KpnI and BamHI sites.

GGATCC: BamHI restriction site.

B-globin 5' UTR.

CCATGG: NcoI restriction site.

Helicase Double Reporter: 24nt Cy3 binding site, 19nt BHQ binding site

CTGCAG: PstI cut site

Nanoluciferase reporter

AAGCTT: HindIII restriction site

Reporter template sequence:

GGTACCTAATACGACTCACTATA**G**GGGGATCCacatttgcttctgacacaactgtgttcactagcaacctcaaacagacaccCTGCAGGAAAAAATTAAAAAATTAAAAAACTCGGAGGGGCCGGTGGGGCCACCATGGTCTTCACACTCGAAGATTTCGTTGGGGACTGGCGACAGACAGCCGGCTACAACCTGGACCAAGTCCTTGAACAGGGAGGTGTGTCCAGTTTGTTTCAGAATCTCGGGGTGTCCGTAACTCCGATCCAAAGGATTGTCCTGAGCGGTGAAAATGGGCTGAAGATCGACATCCATGTCATCATCCCGTATGAAGGTCTGAGCGGCGACCAAATGGGCCAGATCGAAAAAATTTTTAAGGTGGTGTACCCTGTGGATGATCATCACTTTAAGGTGATCCTGCACTATGGCACACTGGTAATCGACGGGGTTACGCCGAACATGATCGACTATTTCGGACGGCCGTATGAAGGCATCGCCGTGTTCGACGGCAAAAAGATCACTGTAACAGGGACCCTGTGGAACGGCAACAAAATTATCGACGAGCGCCTGATCAACCCCGACGGCTCCCTGCTGTTCCGAGTAACCATCAACGGAGTGACCGGCTGGCGGCTGTGCGAACGCATTCTGGCGTAAAAGCTT

Forward primer used to PCR template:

5′-CGTTGTAAAACGACGGCCAG-3′

Reverse primer used to PCR template:

5′-(T)_50_ACCCCAGGCTTTACACTTTATGC-3′

Reverse primer is complementary to the pUC57 sequence downstream of the reporter sequence and adds a 100 nt 3′ UTR sequence from the pUC57 plasmid.

**Supplementary Figure 4**


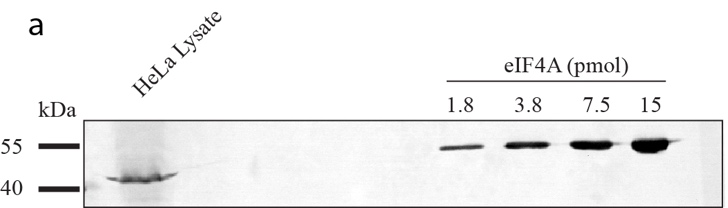


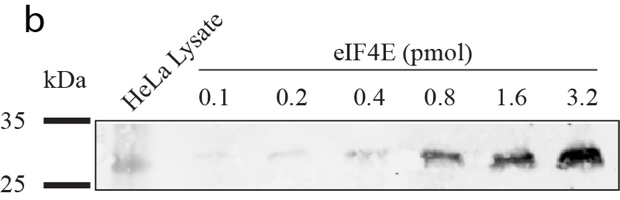


## Figure S4. Quantitation of eIF4E and eIF4A in HeLa cell-free extract. A fixed amount of HeLa cell-free extract (11.5 μl) was analyzed by SDS-PAGE and Western blotting alongside increasing amounts (pmols) of purified recombinant eIF4E (a) or eIF4A (b), as indicated. Western blots were carried out as described in *Experimental Procedures*. (a) An estimation of eIF4E concentration in the HeLa cell-free extract is 44 nM, with a final concentration of 17 nM in the translation assay (which uses 40 % cell-free extract, as described in *Experimental Procedures*). (b) An estimation of eIF4A concentration in the HeLa cell-free extract is 235 nM, with a final concentration of 94 nM in the translation assay (which uses 40 % cell-free extract, as described in *Experimental Procedures*).
